# Baseline predictors of response and discontinuation of tumor necrosis factor-alpha blocking therapy in ankylosing spondylitis: a prospective longitudinal observational cohort study

**DOI:** 10.1186/ar3369

**Published:** 2011-06-20

**Authors:** Suzanne Arends, Elisabeth Brouwer, Eveline van der Veer, Henk Groen, Martha K Leijsma, Pieternella M Houtman, Tim L Th A Jansen, Cees GM Kallenberg, Anneke Spoorenberg

**Affiliations:** 1 Rheumatology and Clinical Immunology, University Medical Center Groningen, University of Groningen, P.O. Box 30.001, 9700 RB Groningen, The Netherlands; 2 Laboratory Medicine, University Medical Center Groningen, P.O. Box 30.001, 9700 RB Groningen, The Netherlands; 3 Epidemiology, University Medical Center Groningen, P.O. Box 30.001, 9700 RB Groningen, The Netherlands; 4 Rheumatology, Medical Center Leeuwarden, P.O. Box 888, 8901 BR Leeuwarden, The Netherlands

## Abstract

**Introduction:**

Identifying ankylosing spondylitis (AS) patients who are likely to benefit from tumor necrosis factor-alpha (TNF-α) blocking therapy is important, especially in view of the costs and potential side effects of these agents. Recently, the AS Disease Activity Score (ASDAS) has been developed to assess both subjective and objective aspects of AS disease activity. However, data about the predictive value of the ASDAS with respect to clinical response to TNF-α blocking therapy are lacking. The aim of the present study was to identify baseline predictors of response and discontinuation of TNF-α blocking therapy in AS patients in daily clinical practice.

**Methods:**

AS outpatients who started TNF-α blocking therapy were included in the Groningen Leeuwarden Ankylosing Spondylitis (GLAS) study, an ongoing prospective longitudinal observational cohort study with follow-up visits according to a fixed protocol. For the present analysis, patients were excluded if they had previously received anti-TNF-α treatment. Predictor analyses of response and treatment discontinuation were performed using logistic and Cox regression models, respectively.

**Results:**

Between November 2004 and April 2010, 220 patients started treatment with infliximab (*n *= 32), etanercept (*n *= 137), or adalimumab (*n *= 51). At three and six months, 68% and 63% of patients were Assessments in Ankylosing Spondylitis (ASAS)20 responders, 49% and 46% ASAS40 responders, and 49% and 50% Bath Ankylosing Spondylitis Disease Activity Index (BASDAI)50 responders, respectively. Baseline predictors of response were younger age, male gender, higher ASDAS score, higher erythrocyte sedimentation rate (ESR) level, higher C-reactive protein (CRP) level, presence of peripheral arthritis, higher patient's global assessment of disease activity, and lower modified Schober test. In August 2010, 64% of patients were still using their TNF-α blocking agent with a median follow-up of 33.1 months (range 2.4 to 68.2). Baseline predictors of discontinuation of TNF-α blocking therapy were female gender, absence of peripheral arthritis, higher BASDAI, lower ESR level, and lower CRP level.

**Conclusions:**

Besides younger age and male gender, objective variables such as higher inflammatory markers or ASDAS score were identified as independent baseline predictors of response and/or continuation of TNF-α blocking therapy. In contrast, higher baseline BASDAI score was independently associated with treatment discontinuation. Based on these results, it seems clinically relevant to include more objective variables in the evaluation of anti-TNF-α treatment.

## Introduction

Randomized controlled trials (RCTs) have demonstrated that the tumor necrosis factor alpha (TNF-α) blocking agents infliximab, etanercept, and adalimumab are effective in the treatment of Ankylosing Spondylitis (AS). However, a significant proportion of patients has to withdraw from TNF-α blocking therapy due to inefficacy or adverse events [1-3]. Identifying patients who are likely to benefit from TNF-α blocking therapy is important, especially in view of the costs and potential side effects of these agents.

Several studies using clinical data from RCTs have focused on the identification of predictors of response to anti-TNF-α treatment in AS [4-6]. However, many patients who are treated with TNF-α blocking therapy in daily clinical practice would have been excluded in RCTs. Until now, three population based registries have investigated predictors of response and/or continuation of TNF-α blocking therapy. These registries showed that raised inflammatory markers, lower Bath Ankylosing Spondylitis Functional Index (BASFI), and younger age at baseline were associated with clinical response [7,8], whereas male gender, raised inflammatory markers, low visual analogue scale (VAS) fatigue, and presence of peripheral arthritis were baseline predictors of longer drug survival [7,9].

Disease activity in AS encompasses a wide range of concepts and is therefore difficult to measure. Recently, the Ankylosing Spondylitis Disease Activity Score (ASDAS) has been developed [10,11]. This new index is a composite score of patient-reported measures and acute phase reactants developed in order to capture both subjective and objective aspects of AS disease activity. Currently, information about the predictive value of the ASDAS with respect to response to TNF-α blocking therapy or drug survival is lacking due to the absence of ASDAS data in previous studies. The aim of the present study was to identify baseline predictors of response and discontinuation of TNF-α blocking therapy in AS patients in daily clinical practice.

## Materials and methods

### Patients

Since 2004 AS outpatients with active disease, who started treatment with the TNF-α blocking agents infliximab, etanercept, or adalimumab at the Medical Center Leeuwarden (MCL) and the University Medical Center Groningen (UMCG), were included in the Groningen Leeuwarden Ankylosing Spondylitis (GLAS) study, an ongoing prospective longitudinal observational cohort study with follow-up visits according to a fixed protocol. All patients were over 18 years of age, fulfilled the modified New York criteria for AS or the Assessments in Ankylosing Spondylitis (ASAS) criteria for axial spondyloarthritis including MRI [12], and started anti-TNF-α treatment because of active disease according to the ASAS consensus statement [13]. For the present analysis, patients were excluded if they had previously received anti-TNF-α treatment. Infliximab (5 mg/kg) was given intravenously at zero, two and six weeks and then every eight weeks. In case of inadequate response, the frequency of infliximab treatment was raised to every six weeks. Etanercept was administered as a subcutaneous injection once (50 mg) or twice (25 mg) a week. Adalimumab (40 mg) was administered as a subcutaneous injection on alternate weeks. In the first years of this study, patients were treated with either infliximab or etanercept since adalimumab was only registered in the Netherlands since 2006. The choice of the TNF-α blocking agent was based on the judgment of the treating rheumatologist (chiefly) and/or the specific preference of the patient. Patients were allowed to receive concomitant medication as usual in daily clinical practice. The study was approved by the local ethics committees of the UMCG and MCL and all patients provided written informed consent according to the Declaration of Helsinki to participate in this study.

### Clinical assessments

Patients were evaluated at baseline, after three and six months of anti-TNF-α treatment, and then every six months. Disease activity was assessed using the Bath Ankylosing Spondylitis Disease Activity Index (BASDAI; on a scale of 0 to 10) [14], physician's and patient's global assessment of disease activity (GDA; on a scale of 0 to 10), erythrocyte sedimentation rate (ESR), C-reactive protein (CRP), and ASDAS calculated from BASDAI questions 2, 3, and 6, patient's GDA, and CRP [10,11]. Physical function was assessed using BASFI (on a scale of 0 to 10) [15]. Spinal mobility assessments included chest expansion, modified Schober test, occiput to wall distance, and lateral lumbar flexion (left and right). Peripheral arthritis was defined as at least one swollen joint (excluding the hip) at baseline.

### Response

At every visit, continuation of treatment was based on a decrease in BASDAI, amounting to at least 50% (BASDAI50 response) or two units compared with baseline, and/or expert opinion in favor of treatment continuation. The ASAS20 and ASAS40 response criteria have been developed for defining treatment response in clinical trials. ASAS20 response was defined as an improvement of at least 20% and absolute improvement of at least one unit (on a scale of 0 to 10) compared with baseline in three or more of the four domains: physical function (BASFI), pain, patient's GDA, and inflammation (mean from BASDAI questions 5 and 6), with no worsening by more than 20% in the remaining domain. ASAS40 response was defined as an improvement of at least 40% and an absolute improvement of at least two units compared with baseline in three or more of the four domains, with no worsening at all in the remaining domain [12,16]. In the present analysis, the ASAS20, ASAS40, and BASDAI50 response criteria were used to define treatment response. Patients who did not respond to TNF-α blocking therapy in the first three months were classified as primary non-responders and patients who lost their initial clinical response as secondary non-responders.

### Antibody assessment

Antibodies to TNF-α blocking agents were measured in patients who discontinued infliximab or adalimumab treatment due to inefficacy. Antibodies were detected by radioimmunoassay (RIA) as described in detail previously [17,18]. The assay measures specific high-avidity IgG antibodies to infliximab or adalimumab by an antigen-binding test. In short, serum (1 μl/test) was pre-incubated with Sepharose-immobilized protein A (1 mg/test; Pharmacia, Uppsala, Sweden) in Freeze buffer (Sanquin, Amsterdam, The Netherlands). Non-bound serum components were removed by washing before 50 μl ^125^I-radiolabeled F(ab)'2 fragment of infliximab or adalimumab was added. After overnight incubation, non-bound radiolabel was washed away and Sepharose-bound radioactivity was measured. Test results were converted into arbitrary units per milliliter (AU/ml) by comparison with dilutions of a reference serum. The reference value was set at 12 AU/ml, as derived from 100 healthy donors.

### Statistical analysis

Statistical analysis was performed with SPSS 16.0 software (SPSS, Chicago, IL, USA). Results were expressed as mean ± SD or median (range) for normally and non-normally distributed data, respectively. The Independent Samples T test and Mann-Whitney U test were used to compare differences between groups. The Chi-Square test and Fisher Exact test were used to compare percentages between groups. Predictor analyses of ASAS20, ASAS40, and BASDAI50 response (yes/no) were performed using binary logistic regression. Predictor analysis of time to discontinuation of TNF-α blocking therapy (yes/no) was performed using Cox regression. Multivariate analysis was performed with conditional stepwise forward inclusion of predictors that had a *P*-value ≤0.3 in the univariate analysis. *P*-values < 0.05 were considered statistically significant.

## Results

Between November 2004 and April 2010, a total of 220 patients (MCL: *n *= 163; UMCG: *n *= 57) started treatment with a first TNF-α blocking agent; 32 receiving infliximab, 137 etanercept, and 51 adalimumab. Mean age of all patients was 42.9 years (SD ± 11.9), median disease duration was 15 years (range 1 to 53), and 69% were male. The three treatment groups were comparable for age, gender, HLA-B27 status, BASDAI, ASDAS, patient's GDA, CRP, ESR, concomitant medication, and presence of peripheral arthritis at baseline. In the infliximab group, time since diagnosis was significantly longer, the percentage of patients with a history of inflammatory bowel disease (IBD) was significantly higher, and occiput to wall distance was significantly larger compared to the etanercept and adalimumab groups. In the adalimumab group, the percentage of patients with a history of uveitis and physician's GDA were significantly lower and chest expansion was significantly higher compared to the infliximab and/or etanercept group (Table 1).

**Table 1:** Baseline characteristics of the AS study population

	Total	IFX	ETA	ADA
Number of patients	220	32	137	51
Age (yrs)	42.9 ± 11.9	45.8 ± 10.1	41.9 ± 11.6	43.7 ± 13.3
Gender (male) (n, %)	152 (69)	20 (63)	96 (70)	36 (71)
Duration of symptoms (yrs)	15 (1 to 53)	21 (2 to 49)	15 (1 to 47)	11 (1 to 53)
Time since diagnosis (yrs)	7 (0 to 45)	16 (0 to 35)*	7 (0 to 44)	6 (0 to 45)
HLA-B27+ (n, %)	174 (81)	24 (75)	108 (82)	42 (82)
History of IBD (n, %)	20 (9)	8 (26)†	7 (5)	4 (8)
History of uveitis (n, %)	64 (29)	13 (40)	44 (32)	7 (14)‡
History of psoriasis (n, %)	13 (6)	3 (9)	8 (6)	13 (6)
Peripheral arthritis (n, %)	37 (17)	5 (16)	27 (20)	5 (10)
Current NSAID use (n, %)	158 (72)	24 (75)	102 (75)	32 (63)
Current DMARD use (n, %)	45 (21)	10 (31)	28 (20)	7 (14)
BASDAI (range 0 to 10)	6.1 ± 1.7	6.1 ± 1.4	6.2 ± 1.7	5.9 ± 1.7
ASDAS	3.8 ± 0.8	3.8 ± 0.6	3.8 ± 0.8	3.7 ± 0.9
Physician's GDA (range 0-10)	5 (0 to 9)	5 (0 to 8)	5 (0 to 9)	3 (0 to 9)‡
Patient's GDA (range 0 to 10)	7 (1 to 10)	6 (1 to 9)	7 (1 to 10)	6 (1 to 10)
ESR (mm/h)	21 (2 to 101)	24 (2 to 90)	20 (2 to 101)	23 (2 to 74)
CRP (mg/l)	13 (2 to 99)	15 (2 to 74)	12 (2 to 99)	14 (2 to 92)
BASFI (range 0 to 10)	6.1 (0.3 to 9.7)	6.3 (1.9 to 9.6)	5.9 (0.3 to 9.7)	6.3 (0.4 to 9.5)
Chest expansion (cm)	3.0 (0.5 to 43.0)	2.5 (0.5 to 7.0)	3.0 (0.5 to 22.0)	3.5 (0.0 to 43.0)§
Modified Schober test (cm)	2.9 (0.0 to 7.0)	2.4 (0.5 to 6.0)	2.8 (0.1 to 7.0)	3.2 (0.0 to 5.5)
Occiput to wall distance (cm)	4.9 (0.0 to 34.5)	9.0 (0.0 to 26.0)^	4.5 (0.0 to 34.5)	3.5 (0.0 to 30.0)
Lateral lumbar flexion L (cm)	8.0 (0.0 to 30.0)	7.0 (2.0 to 15.0)	9.0 (0.0 to 30.0)	8.0 (1.0 to 20.5)
Lateral lumbar flexion R (cm)	8.0 (0.0 to 29.0)	8.0 (0.5 to 17.0)	8.0 (0.0 to 29.0)	7.5 (1.0 to 20.0)

Values are mean ± SD or median (range) unless otherwise indicated.

AS, Ankylosing Spondylitis; IFX, infliximab; ETA, etanercept; ADA, adalimumab; HLA-B27+, human leukocyte antigen B27 positive; IBD, inflammatory bowel disease; NSAID, non-steroidal anti-inflammatory drug; DMARD, disease-modifying antirheumatic drug; BASDAI, Bath Ankylosing Spondylitis Disease Activity Index; ASDAS, Ankylosing Spondylitis Disease Activity Score; GDA, global disease activity; ESR, erythrocyte sedimentation rate; CRP, C-reactive protein; BASFI, Bath Ankylosing Spondylitis Functional Index; L, left; R, right.

* *P *< 0.05 compared to ETA group and *P *= 0.052 compared to ADA group.

† *P *< 0.05 compared to ETA group and *P *= 0.050 compared to ADA group.

‡ *P *< 0.05 compared to IFX and ETA groups.

§ *P *< 0.05 compared to ETA group.

^ *P *< 0.05 compared to ETA and ADA groups.

### ASAS20 response

The percentage of ASAS20 responders to TNF-α blocking therapy was 68% and 63% at three and six months, respectively. No significant differences were found in the percentage of ASAS20 responders between the three TNF-α blocking agents at three or six months (*P *= 0.297 and *P *= 0.128, respectively) (Table 2).

**Table 2:** Response and drug survival rate in AS patients treated with TNF-α blocking therapy

	Total	IFX	ETA	ADA
Number of patients	220	32	137	51
ASAS20 responders at three months	68%	80%	66%	65%
(number of patients)	(145 of 214)	(24 of 30)	(88 of 133)	(33 of 51)
ASAS20 responders at six months	63%	71%	66%	51%
(number of patients)	(132 of 209)	(22 of 31)	(86 of 131)	(24 of 47)
ASAS40 responders at three months	49%	63%	47%	45%
(number of patients)	(104 of 214)	(19 of 30)	(62 of 133)	(23 of 51)
ASAS40 responders at six months	46%	52%	48%	38%
(number of patients)	(97 of 209)	(16 of 31)	(63 of 131)	(18 of 47)
BASDAI50 responders at three months	49%	60%	46%	51%
(number of patients)	(105 of 214)	(18 of 30)	(61 of 133)	(26 of 51)
BASDAI50 responders at six months	50%	48%	51%	47%
(number of patients)	(104 of 209)	(15 of 31)	(67 of 131)	(22 of 47)
One-year drug survival	71%	76%	72%	65%
(number of patients)	(136 of 192)	(22 of 29)	(88 of 123)	(26 of 40)
Two-year drug survival	66%	70%	69%	48%
(number of patients)	(97 of 148)	(19 of 27)	(66 of 96)	(12 of 25)

See Table 1 for definitions.

No statistical differences were found between treatment groups (*P *≥0.05).

Results of univariate and multivariate logistic regression analysis for ASAS20 response at three and six months of anti-TNF-α treatment are presented in Tables 3 and 4, respectively. Male gender (OR: 2.166) was identified as a significant baseline predictor of ASAS20 response in univariate logistic regression analysis. Therefore, variables that significantly differed between men and women at baseline were included in multivariate analysis: age, patient's GDA, ESR, chest expansion, and occiput to wall distance. Multivariate logistic regression analysis showed that younger age (OR: 0.972), male gender (OR: 3.151), and higher ESR level (OR: 1.023) or alternatively, higher CRP level (OR: 1.024) or higher ASDAS score (OR: 1.728), were independent baseline predictors of ASAS20 response at three months of anti-TNF-α treatment (Table 3).

**Table 3:** Baseline predictors of ASAS20 response at three months of anti-TNF-α treatment

		Univariate analysis		Multivariate analysis	
		OR (95% CI)	*P*-value	OR (95% CI)	*P-*value
Age (yr)†^a^		0.982 (0.959 to 1.006)	0.150	0.972 (0.947 to 0.998)	0.035
Gender	Female	1	-		-
	Male	2.166 (1.185 to 3.958)	0.012	3.151 (1.580 to 6.285)	0.001
Duration of symptoms (yr)†		1.001 (0.976 to 1.028)	0.914		***
HLA-B27	Negative	1	-		-
	Positive	0.779 (0.363 to 1.675)	0.523		***
Peripheral arthritis	Absent	1	-		-
	Present	2.120 (0.876 to 5.129)	0.096		**
BASDAI (range 0 to 10)‡		0.946 (0.793 to 1.129)	0.538		***
ASDAS‡		1.458 (0.992 to 2.144)	0.055		*
Physician's GDA (range 0 to 10)‡		1.122 (0.983 to 1.282)	0.089		**
Patient's GDA (range 0 to 10)‡^a^		1.029 (0.882 to 1.201)	0.714		**
ESR (mm/h)‡^a^		1.016 (1.000 to 1.032)	0.049	1.023 (1.005 to 1.041)	0.014
CRP (mg/l)‡		1.021 (1.003 to 1.040)	0.025		*
BASFI (range 0 to 10)‡		0.939 (0.816 to 1.081)	0.382		***
Chest expansion (cm)‡^a^		1.081 (0.948 to 1.233)	0.243		**
Modified Schober test (cm)‡		1.026 (0.861 to 1.224)	0.773		***
Occiput to wall distance (cm)‡^a^		0.981 (0.942 to 1.022)	0.364		**
Lateral lumbar flexion L (cm)‡		1.027 (0.965 to 1.092)	0.402		***
Lateral lumbar flexion R (cm)‡		1.029 (0.968 to 1.094)	0.352		***
TNF-α blocking agent	ETA	1	-		-
	IFX	2.045 (0.780 to 5.364)	0.146		**
	ADA	0.938 (0.476 to 1.846)	0.852		**

See Table 1 for definitions.

OR refers to the risk of achieving ASAS20 response: † per year; ‡ per 1 grade or 1 point.

^a ^Significant difference (*P *< 0.05) between men and women at baseline.

* CRP and ASDAS were not selected during forward conditional logistic regression due to the strong correlation with ESR (ESR and CRP: *ρ *= 0.669, *P *= 0.000; ESR and ASDAS: *ρ *= 0.412, *P *= 0.000). Although, higher CRP level (OR: 1.024, 95% CI: 1.004 to 1.044) and higher ASDAS level (OR: 1.728, 95% CI: 1.126 to 2.652) were also significant predictors of ASAS20 response at three months in the presence of age and gender.

** The variable was not selected during multivariate regression analysis (*P *≥0.05).

*** The variable was not tested in multivariate regression analysis because of a *P*-value > 0.3 in univariate regression analysis and no significant difference between men and women at baseline.

At six months of anti-TNF-α treatment, younger age (OR: 0.960), male gender (OR: 2.991), and higher ASDAS score (OR: 1.573) or alternatively, presence of peripheral arthritis (OR: 2.518) and higher patient's GDA (OR: 1.173), were independent baseline predictors of ASAS20 response (Table 4).

**Table 4:** Baseline predictors of ASAS20 response at six months of anti-TNF-α treatment

		Univariate analysis		Multivariate analysis	
		OR (95% CI)	*P*-value	OR (95% CI)	*P*-value
Age (yr)†^a^		0.977 (0.954 to 1.002)	0.069	0.960 (0.934 to 0.987)	0.004
Gender	Female	1	-		-
	Male	1.995 (1.087 to 3.659)	0.026	2.991 (1.519 to 5.890)	0.002
Duration of symptoms (yr)†		0.997 (0.972 to 1.023)	0.821		***
HLA-B27	Negative	1	-		-
	Positive	1.086 (0.520 to 2.266)	0.827		***
Peripheral arthritis	Absent	1	-		-
	Present	2.218 (0.952 to 5.165)	0.065		*
BASDAI (range 0 to 10)‡		1.031 (0.873 to 1.219)	0.717		***
ASDAS‡		1.356 (0.945 to 1.946)	0.099	1.573 (1.051 to 2.354)	0.028
Physician's GDA (range 0 to 10)‡		1.087 (0.955 to 1.239)	0.207		**
Patient's GDA (range 0 to 10)‡^a^		1.124 (0.973 to 1.300)	0.113		*
ESR (mm/h)‡^a^		1.005 (0.991 to 1.019)	0.499		**
CRP (mg/l)‡		1.009 (0.993 to 1.024)	0.281		**
BASFI (range 0 to 10)‡		0.989 (0.861 to 1.135)	0.872		***
Chest expansion (cm)‡^a^		1.108 (0.953 to 1.289)	0.183		**
Modified Schober test (cm)‡		0.900 (0.755 to 1.074)	0.243		**
Occiput to wall distance (cm)‡^a^		0.989 (0.950 to 1.030)	0.591		**
Lateral lumbar flexion L (cm)‡		0.985 (0.928 to 1.044)	0.606		***
Lateral lumbar flexion R (cm)‡		1.018 (0.960 to 1.079)	0.557		***
TNF-α blocking agent	ETA	1	-		-
	IFX	1.279 (0.544 to 3.008)	0.573		**
	ADA	0.546 (0.278 to 1.076)	0.079		**

See Table 1 for definitions.

OR refers to the risk of achieving ASAS20 response: † per year; ‡ per 1 grade or 1 point.

^a ^Significant difference (*P *< 0.05) between men and women at baseline.

* Presence of peripheral arthritis and patient's GDA were not selected during forward conditional logistic regression due to the significant difference in ASDAS score between patients with and without peripheral arthritis (mean 4.2 vs. 3.7, *P *= 0.001) and the strong correlation between ASDAS and patient's GDA (*ρ *= 0.508, *P *= 0.000). Although, presence of peripheral arthritis (OR: 2.518, 95% CI: 1.053 to 6.025) and higher patient's GDA (OR: 1.173, 95% CI: 1.003 to 1.372) were also significant predictors of ASAS20 response at 6 months in the presence of age and gender.

** The variable was not selected during multivariate regression analysis (*P *≥0.05).

*** The variable was not tested in multivariate regression analysis because of a *P*-value > 0.3 in univariate regression analysis and no significant difference between men and women at baseline.

### ASAS40 response

The percentage of ASAS40 responders to TNF-α blocking therapy was 49% and 46% at three and six months, respectively. No significant differences were found in the percentage of responders between the three TNF-α blocking agents at three or six months (*P *= 0.216 and *P *= 0.421, respectively) (Table 2).

Multivariate logistic regression analysis showed that younger age (OR: 0.970, 95% CI: 0.946 to 0.994) was the only independent baseline predictor of ASAS40 response at three months of anti-TNF-α treatment.

At six months of anti-TNF-α treatment, younger age (OR: 0.961, 95% CI: 0.935 to 0.987), male gender (OR: 2.488, 95% CI: 1.235 to 5.014), and higher patient's GDA (OR: 1.258, 95% CI: 1.067 to 1.483) or alternatively, higher ASDAS score (OR: 1.721, 95% CI: 1.159 to 2.555), were independent baseline predictors of ASAS40 response.

### BASDAI50 response

The percentage of BASDAI50 responders to TNF-α blocking therapy was 49% and 50% at three and six months, respectively. No significant differences were found in the percentage of responders between the three TNF-α blocking agents at three or six months (*P *= 0.358 and *P *= 0.866, respectively) (Table 2).

Multivariate logistic regression analysis showed that younger age (OR: 0.975, 95% CI: 0.951 to 0.999), male gender (OR: 2.572, 95% CI: 1.346 to 4.913), and higher CRP level (OR: 1.025, 95% CI: 1.008 to 1.042) or alternatively, higher ESR level (OR: 1.026, 95% CI: 1.009 to 1.042), were independent baseline predictors of BASDAI50 response at three months of anti-TNF-α treatment.

At six months of anti-TNF-α treatment, younger age (OR: 0.957, 95% CI: 0.929 to 0.985), male gender (OR: 2.598, 95% CI: 1.302 to 5.186), presence of peripheral arthritis (OR: 4.991, 95% CI: 2.054 to 12.124), and lower modified Schober test (OR: 0.751, 95% CI: 0.610 to 0.924) were independent baseline predictors of BASDAI50 response.

### Treatment discontinuation

In August 2010, 141 (64%) patients were still using their TNF-α blocking agent with a median follow-up of 33.1 months (range 2.4 to 68.2). The remaining 79 (36%) patients discontinued TNF-α blocking therapy after median treatment duration of 7.0 months (range 0.2 to 55.6). Reasons for discontinuation of TNF-α blocking therapy were inefficacy (*n *= 40, 51%), adverse events (*n *= 21, 27%: infection (*n *= 8); allergic reaction (*n *= 4); diarrhea or IBD (*n *= 5); cardio-vascular disease (*n *= 2); demyelization problems (*n *= 1); bladder cancer (*n *= 1)), both inefficacy and adverse events (*n *= 8, 10%: recurrent infections (*n *= 3); allergic reaction (*n *= 1); diarrhea or IBD (*n *= 2); uveitis (*n *= 1); malaise (*n *= 1)), or other reasons (*n *= 10, 13%: good initial response, own choice (*n *= 3); pregnancy wish (*n *= 5); lost to follow up (*n *= 2)).

Antibodies to TNF-α blocking agents were measured in patients who discontinued infliximab (*n *= 7) or adalimumab (*n *= 14) treatment due to inefficacy. Antibody data were missing for one adalimumab patient. Antibodies against infliximab and adalimumab were detected in 5 of 7 (71%) and in 8 of 13 (62%) patients who discontinued treatment due to inefficacy, respectively. In total, 5 of 13 (38%) patients with antibodies to TNF-α blocking agents were primary non-responders and 8 of 13 (62%) patients were secondary non-responders.

The one-year and two-year TNF-α blocking therapy survival rates were 71% and 66%, respectively. No significant differences were found in one-year or two-year survival rates between the three TNF-α blocking agents (*P *= 0.593 and *P *= 0.127, respectively) (Table 2).

Results of univariate and multivariate Cox regression analysis for discontinuation of anti-TNF-α treatment are presented in Table 5. Since female gender (HR: 0.503) and absence of peripheral arthritis (HR: 0.382) were significantly associated with treatment discontinuation in univariate Cox regression analysis, baseline variables that significantly differed between men and women (age, patient's GDA, ESR, chest expansion, and occiput to wall distance) or between patients with and without peripheral arthritis (BASDAI, ASDAS, physician's GDA, and CRP) were included in multivariate analysis. Multivariate Cox regression analysis showed that female gender (HR: 0.406), absence of peripheral arthritis (HR: 0.320), higher BASDAI score (HR: 1.225), and lower ESR level (HR: 0.983) or alternatively, lower CRP level (HR: 0.984), were independent baseline predictors of discontinuation of anti-TNF-α treatment (Table 5).

**Table 5:** Baseline predictors of anti-TNF-α treatment discontinuation

		Univariate analysis		Multivariate analysis	
		HR (95% CI)	*P*-value	HR (95% CI)	*P*-value
Age (yr)†^a^		0.994 (0.975 to 1.014)	0.561		**
Gender	Female	1	-		-
	Male	0.503 (0.321 to 0.787)	0.003	0.406 (0.251 to 0.657)	0.000
Duration of symptoms (yr)†		0.981 (0.959 to 1.002)	0.082		**
HLA-B27	Negative	1	-		-
	Positive	0.823 (0.468 to 1.448)	0.500		***
Peripheral arthritis	Absent	1	-		-
	Present	0.382 (0.176 to 0.830)	0.015	0.320 (0.144 to 0.712)	0.005
BASDAI (range 0 to 10)‡^b^		1.162 (1.016 to 1.329)	0.028	1.225 (1.053 to 1.424)	0.008
ASDAS‡^b^		1.005 (0.759 to 1.330)	0.974		**
Physician's GDA (range 0 to 10)‡^b^		0.907 (0.816 to 1.008)	0.070		**
Patient's GDA (range 0 to 10)‡^a^		1.075 (0.958 to 1.208)	0.219		**
ESR (mm/h)‡^a^		0.987 (0.974 to 0.999)	0.039	0.983 (0.969 to 0.997)	0.018
CRP (mg/l)‡^b^		0.986 (0.972 to 1.000)	0.049		*
BASFI (range 0 to 10)‡		1.045 (0.935 to 1.168)	0.438		***
Chest expansion (cm)‡^a^		0.986 (0.903 to 1.076)	0.753		**
Modified Schober test (cm)‡		1.189 (1.036 to 1.365)	0.014		**
Occiput to wall distance (cm)‡^a^		0.971 (0.938 to 1.006)	0.971		**
Lateral lumbar flexion L (cm)‡		1.018 (0.973 to 1.066)	0.434		***
Lateral lumbar flexion R (cm)‡		1.016 (0.971 to 1.062)	0.498		***
TNF-α blocking agent	ETA	1	-		-
	IFX	0.847 (0.441 to 1.627)	0.618		***
	ADA	1.334 (0.769 to 2.314)	0.305		***

See Table 1 for definitions.

HR refers to the risk of anti-TNF-α treatment discontinuation: † per year; ‡ per 1 grade or 1 point.

^a ^Significant difference (*P *< 0.05) between men and women at baseline.

^b ^Significant difference (*P *< 0.05) between patients with peripheral arthritis (defined as at least one swollen joint) and only axial disease at baseline.

* CRP was not selected during forward conditional logistic regression due to the strong correlation with ESR (*ρ *= 0.669, *P *= 0.000) and the significant difference in CRP level between patients with and without peripheral arthritis (median 17 vs. 12, *P *= 0.014). Although, lower CRP level (HR: 0.984, 95% CI: 0.969 to 0.999) was also a significant predictor of treatment discontinuation in the presence of gender and BASDAI.

** The variable was not selected during multivariate regression analysis (*P *≥0.05).

*** The variable was not tested in multivariate regression analysis because of a *P*-value > 0.3 in univariate regression analysis and no significant difference between men and women at baseline.

## Discussion

In this prospective longitudinal observational cohort study, ASAS20 and ASAS40 response was reached by 51% to 80% and 38% to 63% of AS patients at three to six months of anti-TNF-α treatment, respectively. These results from daily practice are in line with the findings in RCTs [1-3]. Although TNF-α blocking therapy is effective in the majority of AS patients, identifying patients who are likely to benefit from TNF-α blocking therapy is important, especially in view of the potential side effects and financial burden of these agents. Data from observational studies are necessary, since inclusion criteria of RCTs are very strict and, therefore, not completely comparable to the criteria for starting TNF-α blocking therapy in daily clinical practice. Our finding that younger AS patients respond significantly better to anti-TNF-α treatment is in line with previous studies using data from RCTs and population based registries [5-7]. Previous studies in rheumatoid arthritis (RA) also found that females were less likely to achieve remission on anti-TNF-α treatment [19,20]. Furthermore, female gender was significantly associated with discontinuation of TNF-α blocking therapy in registries of arthritic rheumatic diseases [21,22] and AS [7,9]. Unfortunately, it is still unclear why male patients respond better to TNF-α blocking therapy.

Multiple studies have shown the importance of raised inflammatory markers with regard to achieving clinical response [4-7] or treatment continuation [7]. This study also confirms the predictive value of high ESR or CRP levels. Our finding that absence of peripheral arthritis is associated with treatment discontinuation is in accordance with Kristensen *et al.*, who reported that patients with peripheral arthritis are more likely to continue TNF-α blocking therapy [9]. In the present study, presence of peripheral arthritis was also independently related to ASAS20 and BASDAI50 response at six months in the presence of age and gender, indicating that concomitant peripheral arthritis is a predictor of both response and continuation of anti-TNF-α treatment.

Recently, the ASDAS has been developed to assess a broader spectrum of disease activity [10,11]. A new and interesting finding is that higher ASDAS score was identified as a significant baseline predictor of ASAS20 and ASAS40 response to TNF-α blocking therapy in this study. Until now, in clinical practice, starting and continuation TNF-α blocking therapy is mainly based on BASDAI response, which is solely based on the opinion of the patient. In this study, more objective variables such as higher inflammatory markers and higher ASDAS score were identified as independent baseline predictors of response and/or continuation of anti-TNF-α treatment. In contrast, a higher baseline BASDAI score was independently associated with treatment discontinuation. Based on these results, it seems clinically relevant to include more objective variables in the evaluation of anti-TNF-α treatment.

Our finding that the majority of AS patients discontinued TNF-α blocking therapy because of inefficacy is in accordance with Glintborg *et al*. [7], but other registries found an almost equal distribution between treatment withdrawal due to adverse events and inefficacy [9,23] or even a higher discontinuation rate because of adverse events [21,22]. These differences may be explained by variation in the classification of reasons for stopping TNF-α blocking therapy.

Since previous studies in AS patients treated with etanercept have reported that no antibodies against etanercept could be detected [24,25], antibodies were only measured in patients who discontinued infliximab and adalimumab due to inefficacy in this study. Antibody formation seems to be related to inefficacy of infliximab and adalimumab since these antibodies were detected in almost two third of patients (13 out of 20) who discontinued infliximab or adalimumab treatment due to inefficacy. This is in line with our previous findings in a smaller group of AS patients. In this study, patients with antibodies had significantly lower serum TNF-α blocker levels compared to patients without antibodies and significant negative correlations between serum levels of TNF-α blocking agents and assessments of disease activity were found [24]. Based on these results, it seems useful to determine antibody formation to TNF-α blocking agents in non-responsive AS patients.

In the present study, we did not find significant differences in the percentage of ASAS20, ASAS40, or BASDAI50 responders at three and six months or in one-year and two-year drug survival rates between the three TNF-α blocking agents. Furthermore, the type of anti-TNF-α treatment (infliximab, etanercept, or adalimumab) was not significantly associated with achieving response or discontinuation of treatment. However, these findings should be interpreted with caution since there were differences in disease duration, the percentage of patients with extra-articular manifestations, physician's GDA, and spinal mobility measures at baseline and there was an uneven distribution of patients among the different treatment groups.

## Conclusions

This prospective longitudinal observational cohort study identified higher ASDAS score, higher ESR or CRP level, presence of peripheral arthritis, younger age, male gender, lower modified Schober test, higher patient's GDA, and lower BASDAI as independent baseline predictors of response and/or continuation of TNF-α blocking therapy in AS patients. These findings may help clinicians to identify AS patients who are more likely to benefit from TNF-α blocking therapy in daily clinical practice.

## Abbrevations

AS: Ankylosing Spondylitis; ASAS: Assessments in Ankylosing Spondylitis; ASDAS: Ankylosing Spondylitis Disease Activity Score; BASDAI: Bath Ankylosing Spondylitis Disease Activity Index; BASFI: Bath Ankylosing Spondylitis Functional Index; CRP: C-reactive protein; ESR: erythrocyte sedimentation rate; GDA: global disease activity; HR: hazard ratio; IBD: inflammatory bowel disease; MCL: Medical Center Leeuwarden; OR: odds ratio; RA: rheumatoid arthritis; RCTs: randomized controlled trials; RIA: radioimmunoassay; TNF-α: tumor necrosis factor alpha; UMCG: University Medical Center Groningen; VAS: visual analogue scale.

## Competing interests

EB has received unrestricted research grants from Abbott, Schering-Plough, and Wyeth. AS has received unrestricted research grants from Abbott and Wyeth. The other authors declare that they have no competing interests.

## Authors' contributions

SA performed the statistical analysis and interpretation of data and drafted the manuscript; EB and AS participated in the design of the study, performed the acquisition of data, and critically revised the manuscript. EV and HG contributed to the statistical analysis and interpretation of data and critically revised the manuscript. ML, PH and TJ contributed to the acquisition of clinical data and critically revised the manuscript. CK participated in the design of the study and critically revised the manuscript. All authors read and approved the final manuscript.
